# Supplementary material for: Content-rich biological network constructed by mining PubMed abstracts
Source: BMC Bioinformatics. 2004 Oct 8;5:147. doi: 10.1186/1471-2105-5-147 (PMC528731; doi:10.1186/1471-2105-5-147)
Supplement: Additional File 2 — The original results of the above study (non-essential files are deleted to keep the file size under the limit set by BMC bioinformatics). [file 1471-2105-5-147-S2.bz2 › chilibotAdditionalFile2/dip30/89ID7962001E326/html/RECO_RECR.html]

 


 **RECO** and **RECR** 
  
Found 42 abstracts in PubMed, retrieved 30.  
 

 What does Google say? 
 PDF only 
| .edu only 

---

**Interactive relationship** (e.g. stimulation, inhibition, etc)

**Neutral relationship**- These data suggested that  **RecO**  mediates the interactions of RecF protein with  **RecR**  and with Ssb proteins.  Ref: 8962075 Proc Natl Acad Sci U S A, 1996
- These data suggest that one role of RecF is to stabilize the interaction of  **RecR**  with  **RecO**  in the presence of Ssb protein.  Ref: 8962075 Proc Natl Acad Sci U S A, 1996
- Incubation of RecF,  **RecO** ,  **RecR** , and Ssb proteins resulted in the formation of RecF  **RecO**  Ssb complexes ; i.e.,  **RecR**  was excluded.  Ref: 8962075 Proc Natl Acad Sci U S A, 1996
- Interactions of RecF protein with  **RecO** ,  **RecR** , and single stranded DNA binding proteins reveal roles for the RecF  **RecO**   **RecR**  complex in DNA repair and recombination.  Ref: 8962075 Proc Natl Acad Sci U S A, 1996
- We therefore assigned  **recO**  to epistatic group alpha and predict that the  **RecO**  protein acts at the same stage of recombination as the RecF, RecL and  **RecR**  proteins, in a RecFLOR complex.  Ref: 10323239 Mol Gen Genet, 1999
- Preincubation of RecF,  **RecO** , and  **RecR**  proteins prior to addition of Ssb protein resulted in the formation of complexes consisting of RecF,  **RecO** ,  **RecR** , and Ssb proteins.  Ref: 8962075 Proc Natl Acad Sci U S A, 1996
- It is inferred from these results that recF overexpression may reduce UV inducible sulAp expression by a mechanism that is sensitive to the ability of RecF to bind ATP and to the levels of  **RecO**  and  **RecR**  RecOR in the cell, but not to the type of DNA damage per se.  Ref: 7830722 Mol Gen Genet, 1994
- On the other hand, the presence of recA2020, a suppressor mutation for recF,  **recO** , and  **recR**  mutations, restored normal UVM in recF,  **recO** , and  **recR**  mutants.  Ref: 9537373 J Bacteriol, 1998

**Non-interactive relationship** (e.g. studied together, co-existance, homology, etc.)

- **recO**  and  **recR**  mutations delay induction of the SOS response in Escherichia coli.  Ref: 7862097 Mol Gen Genet, 1995
- These results indicate an involvement of the recF,  **recO** , and  **recR**  genes and their products in UVM, possibly by affecting the third role of RecA in UVM.  Ref: 9537373 J Bacteriol, 1998
- RecF protein competes with  **RecO**  protein for  **RecR**  protein association and its addition destabilizes RecAOR filaments.  Ref: 11743007 EMBO J, 2001
- We analyzed effects of overexpression of RecE and RecT on illegitimate recombination during prophage induction in Escherichia coli and found that frequencies of spontaneous and UV induced illegitimate recombination are enhanced by coexpression of RecE and RecT in the wild type, but the enhanced recombination was reduced by recJ,  **recO** , or  **recR**  mutation.  Ref: 12169595 J Bacteriol, 2002
- The newly formed filaments contain an amount of RecA protein and exhibit an ATP hydrolysis rate comparable to that observed when the  **RecO**  and  **RecR**  proteins are included in the initial reaction mixture.  Ref: 9048946 J Mol Biol, 1997
- RecF,  **RecO**  and  **RecR** , three of the important proteins of the RecF pathway of recombination, are also needed for repair of DNA damage due to UV irradiation.  Ref: 7862097 Mol Gen Genet, 1995
- RecA protein filaments end dependent dissociation from ssDNA and stabilization by  **RecO**  and  **RecR**  proteins.  Ref: 9048946 J Mol Biol, 1997
- In Escherichia coli, the recovery of replication following UV induced DNA damage is dependent upon several proteins in the recF pathway, including RecF,  **RecO** , and  **RecR** .  Ref: 10589843 Mol Gen Genet, 1999
- In addition, we show that  **recO**  and  **recR**  mutations delay induction of beta galactosidase activity expressed from a lexA regulated promoter following exposure of cells to UV, thus further supporting the idea that recF,  **recO**  and  **recR**  gene products are needed for induction of the SOS response.  Ref: 7862097 Mol Gen Genet, 1995
- Gap repair genes recF,  **recO** , and  **recR**  were not required.  Ref: 10224240 Genetics, 1999
- The protective effect of plasmid pGam18 against radiation was completely abolished by mutations in the most RecF pathway genes recF, recJ,  **recR** ,  **recO** , recQ, recN, and ruvB.  Ref: 10368783 Genetika, 1999
- However, lexA Def  **recO**  or lexA Def  **recR**  strains, although unable to facilitate PriA pol II dependent restart, were able to perform pol V dependent readthrough.  Ref: 11929519 Mol Microbiol, 2002
- Several lines of evidence suggest that the recF,  **recO** , and  **recR**  genes function at the same step of recombination and postreplication repair.  Ref: 9537373 J Bacteriol, 1998
- We discuss these results to explain how the RecF  **RecO**   **RecR**  complex functions as an anti Ssb factor.  Ref: 8962075 Proc Natl Acad Sci U S A, 1996
- The  **recO**  mutation did not affect the sensitivity of recF,  **recR**  or recL cells to DNA damaging agents, increased the sensitivity of recU and addAB cells and abolished the DNA repair capacity of recH cells.  Ref: 10323239 Mol Gen Genet, 1999
- Roles for lambda Orf and Escherichia coli  **RecO** ,  **RecR**  and RecF in lambda recombination.  Ref: 9335578 Genetics, 1997
- Expression of the Escherichia coli mutA mutator phenotype requires recA, recB, recC, ruvA, and ruvC gene, but not recD, recF,  **recO** , or  **recR**  genes.  Ref: 10671469 J Bacteriol, 2000
- In this communication, by measuring the in vivo levels of LexA repressor using anti LexA antibodies, we show that  **recO**  and  **recR**  mutant strains are also not proficient in LexA cleavage reactions.  Ref: 7862097 Mol Gen Genet, 1995
- This function is countered by the RecF  **RecO**  competition for association with the  **RecR**  protein.  Ref: 11743007 EMBO J, 2001
- Resistance of E. coli strains to cisplatin as determined by plating experiments decreased in the following order parent PQ30 strain, polB >  **recO** ,  **recR** , recF > recB.  Ref: 12531023 DNA Repair Amst , 2002
- In this work, we report that null mutations in recF,  **recO** , or  **recR**  greatly reduce UV radiation mutagenesis UVM in an assay for reversion from a Trp trpE65 to a Trp phenotypes.  Ref: 9537373 J Bacteriol, 1998
- The Escherichia coli RecF,  **RecO**  and  **RecR**  pro teins have previously been implicated in bacterial recombinational DNA repair at DNA gaps.  Ref: 11743007 EMBO J, 2001
- It was found that recF overexpression reduced sulAp expression after DNA damage by mitomycin C or nalidixic acid,  **recO**  and  **recR**  overexpression partially suppressed the reduction of UV induced sulAp expression caused by recF overexpression.  Ref: 7830722 Mol Gen Genet, 1994
- When the  **RecO**  and  **RecR**  proteins are added after most of the RecA protein has already dissociated, RecA protein filaments re form after a short lag.  Ref: 9048946 J Mol Biol, 1997
- ii In a recBC sbcA background, in which RecE mediated recombination is active, recA, recJ, recQ, recE, recT, recF,  **recO** , and  **recR**  mutations led to their accumulation.  Ref: 12718760 BMC Mol Biol< MedlineTA>BMC Mol Biol, 1997
- Genetic evidence suggests that the RecF,  **RecO** , and  **RecR**  RecFOR proteins participate in a common step of DNA recombination and repair, yet the biochemical event requiring collaboration of all three proteins is unknown.  Ref: 12769856 Mol Cell, 2003
- We show that normal induction also requires the products of  **recO**  and  **recR** .  Ref: 7862088 Mol Gen Genet, 1995
- Introduction of the defective lexA51 mutation lexA51 Def and or UmuD into recF,  **recO** , and  **recR**  mutants failed to restore normal UVM in the mutants.  Ref: 9537373 J Bacteriol, 1998
- Disassembly is not observed even with ATP when both the  **RecO**  and  **RecR**  proteins are present in the initial reaction mixture.  Ref: 9048946 J Mol Biol, 1997
- Involvement of recF,  **recO** , and  **recR**  genes in UV radiation mutagenesis of Escherichia coli.  Ref: 9537373 J Bacteriol, 1998
- Although the MRF alpha 2 components are apparently not encoded by currently known homologous recombination genes such as recA, recF,  **recO** , and  **recR** , they may fulfill an important function in assembling replisomes on arrested replication forks and products of homologous strand exchange.  Ref: 11459960 Proc Natl Acad Sci U S A, 2001
- dnaC suppressors of priA overcome this inviability, especially when RecF,  **RecO**  or  **RecR**  is inactivated, indicating that RdgC avoids or counters a toxic effect of these proteins.  Ref: 12554673 EMBO J, 2003
- Roles of RecJ,  **RecO** , and  **RecR**  in RecET mediated illegitimate recombination in Escherichia coli.  Ref: 12169595 J Bacteriol, 2002
- These observations are incorporated into an expanded model for the functions of RecF,  **RecO** , and  **RecR**  proteins in the early stages of postreplication DNA repair.  Ref: 9363943 Cell, 1997
- Different DNA damaging agents and amounts of  **RecO**  and  **RecR**  were tested for their effects on this phenotype.  Ref: 7830722 Mol Gen Genet, 1994
- Models for the roles of Orf and  **RecO** ,  **RecR**  and RecF in recombination are presented.  Ref: 9335578 Genetics, 1997
- ii this pathway s requires functional recA, recF,  **recO** , and  **recR**  genes, suggesting the mechanism to be daughter strand gap repair.  Ref: 11073901 J Bacteriol, 2000
- Expression of a gene s from the nin region of phage lambda partially complemented both the viability and recombination defects of the recF,  **recO** , and  **recR**  mutants and the recombination defect of ruvC but not of ruvAB or recQ mutants.  Ref: 10735883 J Bacteriol, 2000
- The results indicated that RecET mediated illegitimate recombination depends on the functions of RecJ,  **RecO** , and  **RecR** , suggesting that the RecE and RecJ exonucleases play different roles in this recombination pathway.  Ref: 12169595 J Bacteriol, 2002
- **RecR**  protein, which is known to stimulate  **RecO**  protein to facilitate SSB protein displacement by RecA protein, inhibits annealing by  **RecO**  protein, suggesting that  **RecR**  protein may regulate the choice between the DNA strand invasion versus annealing pathways.  Ref: 12438681 Proc Natl Acad Sci U S A, 2002
- A  **recO**  deficient strain did not exhibit dramatic reduction in efficiency of IRR, implying that RecF and  **RecR**  proteins maintain function during this mechanism in the absence of functional  **RecO** .  Ref: 8921869 Gene, 1996
- Finally, examples are shown for in vitro reactions involving DNA, RecA,  **RecO** , RecF,  **RecR** , and SSB that lead to a further understanding of recombinational repair.  Ref: 11098470 Mol Biotechnol, 2000
- The  **RecO**  and  **RecR**  proteins thereby stabilize RecA filaments even at the 5 ends of ssDNA, a fact which should affect the recombination potential of 5 ends relative to 3 ends.  Ref: 9048946 J Mol Biol, 1997
- Furthermore, we find that  **RecO**  and  **RecR**  significantly stimulate this recombination directed DNA replication reaction,.  Ref: 12667462 Mol Cell, 2003
- The recF,  **recO** , and  **recR**  genes were originally identified as those affecting the RecF pathway of recombination in Escherichia coli cells.  Ref: 9537373 J Bacteriol, 1998
- The products of the recF,  **recO** , and  **recR**  genes are thought to interact and assist RecA in the utilization of single stranded DNA precomplexed with single stranded DNA binding protein Ssb during synapsis.  Ref: 8962075 Proc Natl Acad Sci U S A, 1996
- The defects in restart attributed to mutations in recF,  **recO**  or  **recR**  were suppressed in a recA730 lexA Def strain expressing constitutively activated RecA RecA\*.  Ref: 11929519 Mol Microbiol, 2002
- We compared the recombination proficiencies of recB1080,  **recO** ,  **recR** , and recF single mutants and recB1080  **recO** , recB1080  **recR** , and recB1080 recF double mutants.  Ref: 12618388 Genetics, 2003
- Similarly, recF,  **recO**  or  **recR**  mutations suppress the lethality of the Escherichia coli rep uvrD double mutant.  Ref: 12065426 EMBO J, 2002
- the  **RecO**  and  **RecR**  proteins also play important roles in the recombination.  Ref: 12169595 J Bacteriol, 2002
- In a wild type, lexA background, mutations in recF,  **recO**  or  **recR**  block both pathways.  Ref: 11929519 Mol Microbiol, 2002
- To elucidate the role of several key proteins involved in this process, we have analysed the time dependence of replication restart in strains carrying a combination of mutations in lexA, recA, polB pol II, umuDC pol V, priA, dnaC, recF,  **recO**  or  **recR** .  Ref: 11929519 Mol Microbiol, 2002
- Since recF is epistatic with  **recO**  and  **recR**  recOR for UV resistance, recOR mutations were also tested with priA2 kan.  Ref: 8820655 Mol Microbiol, 1996
- previously isolated mutations in B.subtilis recF, recL,  **recO**  and  **recR** , which belong to the same complementation group, all suppress the lethality of a pcrA mutation.  Ref: 12065426 EMBO J, 2002
- Induction was delayed to a similar degree by mutation in recF,  **recO**  or  **recR** .  Ref: 7862088 Mol Gen Genet, 1995
- Bacteriophage lambda lacking its Red recombination functions requires either its own gene product, Orf, or the product of Escherichia coli s  **recO** ,  **recR**  and recF genes RecORF for efficient recombination in recBC sbcB sbcC mutant cells the RecF pathway.  Ref: 9335578 Genetics, 1997
- An enhanced function of the  **RecO**  and  **RecR**  proteins can thus be seen in vitro at the 5 ends of linear ssDNA that is not as evident in DNA gaps.  Ref: 11743007 EMBO J, 2001
- Altered SOS induction associated with mutations in recF,  **recO**  and  **recR** .  Ref: 7862088 Mol Gen Genet, 1995
- Using immunoprecipitation, size exclusion chromatography, and Ssb protein affinity chromatography in the absence of any nucleotide cofactors, we have obtained the following results i RecF interacts with  **RecO** , ii RecF interacts with  **RecR**  in the presence of  **RecO**  to form a complex consisting of RecF,  **RecO** , and  **RecR**  RecF  **RecO**   **RecR** .  Ref: 8962075 Proc Natl Acad Sci U S A, 1996
- In this genetic background, mutations in recF,  **recO** , and  **recR**  had large effects on both cell viability and on recombination.  Ref: 10735883 J Bacteriol, 2000
